# Characteristics of Cardiomyopathy in Patients With Chronic Left Bundle Branch Block Undergoing Right Ventricular Pacing

**DOI:** 10.1111/pace.70020

**Published:** 2025-08-08

**Authors:** Temidayo A. Abe, Favour Markson, Daniel J. Friedman, Larry R. Jackson

**Affiliations:** ^1^ Division of Cardiology Department of Medicine Vanderbilt University Medical Center Nashville Tennessee USA; ^2^ Division of Cardiology Department of Medicine Jefferson Health‐Einstein Hospital Philadelphia Pennsylvania USA; ^3^ Division of Cardiology Department of Medicine Duke University School of Medicine Duke Clinical Research Institute Durham North Carolina USA

## Abstract

**Background:**

Left bundle branch block (LBBB) and right ventricular pacing (RVP) are associated with abnormal myocardial mechanics and cardiomyopathy. Consequently, chronic LBBB may increase the risk of heart failure and mortality in patients undergoing RVP.

**Methods:**

Using the TriNetX Analytics Network database, we identified patients who underwent pacemaker implantation between January 1, 2014 and January 1, 2024. Exclusion criteria included a history of heart failure, previous cardiac devices, cardiac resynchronization therapy (CRT) during the index hospitalization, or a left ventricular ejection fraction (LVEF) of less than 50%. The primary outcome incident systolic heart failure and all‐cause mortality occurring from the index hospitalization through November 2024.

**Results:**

Among 70,526 patients undergoing RVP implantation, 3916 (5.6%) had chronic LBBB prior to the procedure, with a median age of 75 ± 15 years. Over a median follow‐up of 2.5 years, 5356 (7.6%) developed incident systolic heart failure, and 9714 (13.7%) experienced all‐cause mortality. After propensity score matching, chronic LBBB was associated with a higher risk of systolic heart failure (HR: 1.39; 95% CI: 1.20–1.62) but not all‐cause mortality (HR: 0.93; 95% CI: 0.83–1.06). Patients with chronic LBBB who developed systolic heart failure were more likely to present with moderately depressed LVEF and require CRT upgrades during follow‐up compared to those without chronic LBBB.

**Conclusion:**

Chronic LBBB was associated with a higher risk of systolic heart failure, worse left ventricular function, and greater likelihood of CRT upgrade among patients undergoing RVP.

## 1 Background

Right ventricular pacing (RVP) is associated with abnormal left ventricular mechanics, along with molecular and cellular changes that drive adverse ventricular remodeling and the development of pacing‐induced cardiomyopathy (PICM). PICM is typically defined by a ≥10% reduction in left ventricular ejection fraction (LVEF) or an absolute LVEF <50%, after ruling out other potential causes of cardiomyopathy [1, 2, 3, 4]. With a reported prevalence of 6%–20% occurring within 1–15 years after device implantation, recent research has focused on identifying risk factors to guide clinical decision‐making and mitigate the risk of PICM [1, 2, 5]. While a high RVP burden (>20%) is considered the most significant risk factor for PICM, recent studies have identified additional predictors of risk, particularly markers of subclinical myocardial dysfunction [5, 6, 7, 8, 9]. These include abnormal global longitudinal strain assessed by speckle‐tracking echocardiography and electrocardiographic evidence of inter‐ or intra‐ventricular conduction delays, such as prolonged intrinsic QRS duration (QRSd >155 ms) and the presence of bundle branch block [5, 7, 8]. These factors have been shown to significantly increase the likelihood of developing PICM, emphasizing the complex interplay between conduction abnormalities and ventricular function.

Chronic left bundle branch block (LBBB) is independently associated with significant alterations in left ventricular mechanics, including mechanical dyssynchrony and molecular remodeling, which parallel the adverse effects of right ventricular pacing (RVP) [10, 11, 12, 13]. As a stand‐alone clinical entity, LBBB contributes to the development of cardiomyopathy and is linked to poor clinical outcomes [14, 15, 16]. When RVP is applied in the setting of chronic LBBB, it may result in a compounded “dyssynchrony on dyssynchrony” phenomenon, amplifying the risk of adverse ventricular remodeling, PICM, and LBBB‐associated cardiomyopathy [1, 5]. This compounded risk is underscored by evidence from one study showing that chronic LBBB is associated with an over 4‐fold higher likelihood of developing PICM compared to patients without LBBB [5]. Despite these findings, the interplay between RVP and cardiomyopathy risk in individuals with chronic LBBB remains poorly characterized and inadequately understood. In this study, we examined the incidence and characteristics of cardiomyopathy and all‐cause mortality in patients undergoing RVP, with and without chronic LBBB.

## 2 Methods

### 2.1 Data Source

This study leveraged data from the TriNetX Analytics Network database, a global federated health research network that compiles de‐identified information from electronic health records (EHRs) of patients across numerous healthcare organizations (HCOs), primarily in the United States. The database is publicly available to licensed users through the TriNetX platform. During the study period, the network encompassed data from over 120 US‐based HCOs, representing more than 250 million patients [17]. The database offers access to detailed patient‐level information in an aggregated and anonymized format, including demographics, diagnoses, procedures, medications, laboratory results, and other clinical data. These data are standardized using International Classification of Diseases (ICD), procedure coding system (PCS), and clinical modification (CM) codes, Current Procedural Terminology (CPT) codes, Logical Observation Identifiers Names and Codes (LOINC), and TriNetX‐specific derivatives.

Each data element contains unique patient and encounter identifiers along with corresponding dates and times, enabling the use of advanced analytics tools for cohort selection, matching, and the analysis of event incidence, prevalence, and comparative outcomes. The dataset is dynamically updated, with over 80% of participating HCOs refreshing their data every 1, 2, or 4 weeks, ensuring that the information remains timely and comprehensive [17].

The use of de‐identified data complies with the HIPAA Privacy Rule, and the study protocol was exempted from Institutional Review Board review due to the anonymized nature of the data.

### 2.2 Study Cohort

Using the TriNetX Analytics Network database, we identified patients who underwent de novo implantation of either leadless or transvenous pacemakers between January 1, 2014 and January 1, 2024, based on ICD‐10‐PCS codes (Table S1). Patients with a history of heart failure, LVEF <50%, an existing cardiac device or prior cardiac device implantation, or placement of a cardiac resynchronization therapy (CRT) device during the index admission were excluded. Patients with chronic LBBB were identified using both ICD‐10‐CM and LOINC codes from encounters occurring prior to the device implantation. Additionally, LOINC codes were utilized to determine QRS duration (QRSd) from electrocardiogram data.

### 2.3 Study Outcomes

The primary outcome was composite systolic heart failure, defined as either a new diagnosis of HFrEF or a decline in LVEF to <50% during follow‐up, based on ICD‐CM codes, TriNetX‐specific derivative codes, echocardiographic data, or all‐cause mortality.

The secondary outcomes included incident systolic heart failure and all‐cause mortality as individual endpoints, along with presumed dyssynchrony‐mediated cardiomyopathy. To identify patients with dyssynchrony‐mediated cardiomyopathy, we excluded those with LBBB who developed myocardial infarction requiring percutaneous coronary intervention or coronary artery bypass grafting, alcohol use disorders, myocarditis, takotsubo cardiomyopathy, hypothyroidism, hyperthyroidism, or substance use disorders during follow‐up from the primary cohort. Among the remaining LBBB patients, individuals who developed incident heart failure were classified as having dyssynchrony‐mediated cardiomyopathy.

This approach enabled the accurate identification of systolic heart failure unrelated to secondary causes. The term “dyssynchrony‐mediated cardiomyopathy” was chosen to acknowledge the significant overlap between LBBB‐induced and pacing‐induced cardiomyopathy. Differentiating these conditions is inherently complex and often unclear, requiring detailed, granular data beyond the scope of our dataset.

Additionally, the secondary outcomes encompassed clinical characteristics of systolic heart failure, including the absolute decline in LVEF, mean increases in N‐terminal pro‐B‐type natriuretic peptide (NT‐proBNP), and management strategies, such as the initiation of guideline‐directed medical therapy for heart failure and device upgrades to CRT, compared between patients with and without chronic LBBB.

### 2.4 Statistical Analysis

The Rao‐Scott chi‐square test was used to compare categorical variables, presented as frequencies (percentages), while continuous variables were analyzed using Student's *t*‐test for means ± standard deviations or medians with interquartile ranges. To address baseline differences between cohorts, propensity score matching was performed using a 1:1 nearest neighbor matching algorithm with a caliper width of 0.1 times the pooled standard deviations for covariates listed in Table 1. The balance of our match was assessed using a propensity score density plot, which displayed an adequate overlap in propensity scores after matching (Figure S1). Risk differences with 95% confidence intervals (CI) were estimated using logistic and linear regression, respectively, within the matched cohorts.

**TABLE 1:** Baseline characteristics of patients undergoing pacemaker implantation with and without preexisting left bundle branch block.

	Before PS matching		After PS matching	
	LBBB (*n* = 3916)	No LBBB (*n* = 66,610)	LBBB (*n* = 3916)	No LBBB (*n* = 3916)
Characteristics				
Age, mean ± SD	76 ± 11	74 ± 13	76 ± 11	76 ± 11
Sex				
Male	53.4	54.1	53.4	53.1
Female	43.7	43.3	43.7	44.2
Race				
White	76.5	72.8	76.5	78.1
Black	4.5	6.6	4.5	3.7
Asian	2.7	3.6	2.7	2.5
Others	13.4	13.4	13.4	13.6
Ethnicity				
Hispanic or Latino	30.1	32.1	30.1	29.6
Comorbidities				
Diabetes mellitus	27.2	21.0	27.2	25.8
Hypertension	76.1	57.0	76.1	76.2
Hypothyroidism	18.1	12.6	18.1	17.2
Hyperthyroidism	1.7	1.2	1.7	1.3
Ischemic heart disease	41.4	27.4	41.4	40.1
Prior PCI	7.2	4.3	7.2	6.3
Prior CABG	6.1	4.7	6.1	5.2
Atrial fibrillation/flutter	29.3	31.4	29.3	29.3
Pacing Indication				
Sinus node dysfunction	35.0	47.0	33.7	46.4
Atrioventricular block	24.6	17.2	24.3	16.1
Complete heart block or Atrioventricular nodal ablation	48.3	30.1	48.2	30.3
Pacing modality				
Transvenous pacemaker	95.1	95.0	95.1	94.8
Leadless pacemaker	4.9	5.0	4.9	5.2
Body mass index, mean ± SD	28.6 ± 6	28.5 ± 6	28.6 ± 6	28.3 ± 6
Laboratory				
Creatinine, mean ± SD	1.5 ± 6.9	1.2 ± 3.7	1.5 ± 6.9	1.2 ± 3.0
Hemoglobin A1c, mean ± SD	6.2 ± 1.4	6.2 ± 1.4	6.2 ± 1.4	6.2 ± 1.4
LVEF, mean ± SD	61.7 ± 7.1	62.2 ± 7.1	61.7 ± 7.1	62.8 ± 7.5
NT‐proBNP pg/mL, mean ± SD	327 ± 625	437 ± 1293	327 ± 625	339 ± 754

*Note*: Data are presented as mean ± standard deviation (SD) for continuous variables and percentages for categorical variables.

Abbreviations: CABG, coronary artery bypass graft; CVD, cardiovascular disease; LBBB, left bundle branch block; LVEF, left ventricular ejection fraction; NT‐proBNP, N‐terminal pro‐B type natriuretic peptide; PCI, percutaneous coronary intervention; PS, propensity score; SD, standard deviation.

Survival analyses were illustrated with Kaplan–Meier curves and compared using log‐rank tests, with *p* values <0.05 considered statistically significant. Sensitivity analyses focused on three subpopulations with dyssynchrony‐mediated cardiomyopathy: those implanted with leadless versus transvenous pacemakers, stratification by pacing indication, and stratification by QRS duration.

All statistical analyses were conducted using the TriNetX Analytics platform's cloud computing resources. TriNetX provided a clinical feasibility and analytics team to assist with study design and query optimization. Missing data were handled by limiting analyses to patients with available data for specific variables. For example, analyses requiring follow‐up echocardiography data were restricted to those with these data points. TriNetX does not impute missing values, which prevents introducing bias from estimation methods.

## 3 Results

### 3.1 Baseline Characteristics

During the study period from January 1, 2014 to January 1, 2024, we identified 70,526 patients who underwent either leadless or transvenous pacemaker implantation and met the inclusion criteria. Among these, 3916 (5.6%) had chronic LBBB. Baseline sociodemographic, hospital characteristics, laboratory, and echocardiographic data for these patients, both before and after propensity score matching, are summarized in Table 1. The mean age was 75 ± 15 years, with the majority being male (54%) and of White race (75%).

Before matching, patients with chronic LBBB were older and had a higher comorbidity burden compared to those without chronic LBBB, including higher rates of hypertension, diabetes mellitus, hypothyroidism, and chronic ischemic heart disease. Additionally, the chronic LBBB group exhibited slightly lower baseline LVEF and higher serum creatinine levels, indicating worse baseline renal function relative to those without chronic LBBB. After 1:1 propensity score matching, baseline characteristics, including comorbidity burden, laboratory values, and echocardiographic data, were comparable between the two groups (Figure S1).

With regards to pacing indication, those with LBBB were more likely to have atrioventricular block or complete heart block as the pacing indication, whereas sinus node dysfunction was more common in those without LBBB (Table 1). These differences persisted after propensity score matching.

### 3.2 Incident Systolic Heart Failure and All‐Cause Mortality

During a median follow‐up period of 2.5 years, there were 5356 (7.6%) cases of incident systolic heart failure and 9714 (13.7%) cases of all‐cause mortality following RVP implantation. Patients with chronic LBBB exhibited higher unadjusted crude rates of composite systolic heart failure or mortality (24.0% vs. 19.0%; *p* < 0.001), systolic heart failure (11.5% vs. 7.4%; *p* < 0.001), and all‐cause mortality (15.0% vs. 13.7%; *p* = 0.018) compared to those without chronic LBBB (Table S2).

**FIGURE 1:**
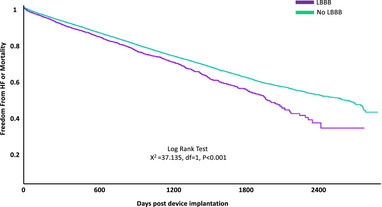
Kaplan–Meier survival curve depicting freedom from the development of composite systolic heart failure (HF) or mortality over time (in days post‐implantation) in patients with chronic LBBB and those without chronic LBBB following RVP implantation. [Colour figure can be viewed at wileyonlinelibrary.com]

**FIGURE 2:**
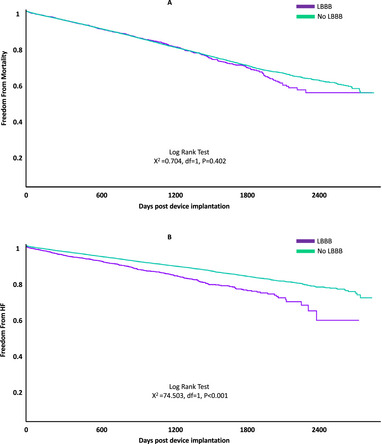
Kaplan–Meier survival curve depicting freedom from the development of mortality (A) and systolic heart failure (HF) (B) over time (in days post‐implantation) in patients with chronic LBBB and those without chronic LBBB following RVP implantation. [Colour figure can be viewed at wileyonlinelibrary.com]

In the adjusted analysis (following propensity score matching), chronic LBBB was associated with approximately 10% higher risk of composite mortality or systolic heart failure (Hazard Ratio [HR]: 1.13, 95% CI: 1.02–1.25) and 40% higher risk of systolic heart failure alone (HR: 1.39, 95% CI: 1.20–1.62). There was no significant difference in all‐cause mortality between the groups in the adjusted analysis (Table 2, Figures 1 and 2).

**TABLE 2:** Clinical outcomes after pacemaker implantation in patients with and without preexisting LBBB after propensity matching.

	LBBB (*n* = 3916)	No LBBB (*n* = 3916)	HR (95% CI)	*p* value
Composite mortality or heart failure	24.0	21.9	1.13 (1.02–1.25)	0.026
New onset systolic heart failure	11.5	8.6	1.39 (1.20–1.62)	<0.001
All‐cause mortality	15.0	15.9	0.93 (0.83–1.06)	0.274

*Note*: Data are presented in percentages. The hazard ratio, 95% confidence interval (CI), and corresponding *p* values for comparisons between the two groups are included.

Abbreviations: CI, confidence interval; LBBB, left bundle branch block.

Importantly, patients with RVP and chronic LBBB who developed systolic heart failure exhibited significantly lower LVEF and were nearly twice as likely to have moderately depressed left ventricular systolic dysfunction, defined as LVEF <40% based on echocardiographic data (HR: 1.82, 95% CI: 1.26–2.65). Additionally, they were two times more likely to require a CRT upgrade during follow‐up compared to those without chronic LBBB (HR: 2.47, 95% CI: 1.80–3.37) (Table 3).

**TABLE 3:** Clinical characteristics of systolic heart failure following pacemaker implantation in patients with and without preexisting LBBB.

	LBBB (*n* = 452)	No LBBB (*n* = 335)	HR (95% CI)	*p* value
LVEF, mean ± SD	45.9 ± 11	47.4 ± 11	1.31 (1.05–1.65)	0.019
LVEF < 40%, (%)	11.2	6.7	1.82 (1.26–2.65)	0.001
NT‐proBNP pg/mL, mean ± SD	1306 ± 2772	1632 ± 4863	0.97 (0.84–1.12)	0.692
Any GDMT (%)	94.0	89.8		0.016
Beta blockers	89.6	84.5		
ARNI, ACE‐I, ARB	95.3	84.2		
MRA	26.2	26.7		
SGLT2‐I	23.7	16.5		
Upgrade to CRT (%)	8.6	3.7	2.47 (1.80–3.37)	<0.001

*Note*: Data are presented as mean ± standard deviation (SD) for continuous variables and percentages for categorical variables. The risk ratio, 95% confidence interval (CI), and corresponding *p* values for comparisons between the two groups are included.

Abbreviations: ACE‐I, angiotensin‐converting enzyme inhibitor; ARB, angiotensin receptor blocker; ARNI, angiotensin receptor‐neprilysin inhibitor; CRT, cardiac resynchronization therapy; GDMT, guideline‐directed medical therapy; HR, hazard ratio; LVEF, left ventricular ejection fraction; MRA, mineralocorticoid receptor antagonist; NT‐proBNP, N‐terminal pro‐B‐type natriuretic peptide; SD, standard deviation; SGLT2‐I, sodium‐glucose cotransporter‐2 inhibitor.

### 3.3 Incident Dyssynchrony‐Mediated Cardiomyopathy

After excluding patients with LBBB who had the secondary causes previously outlined from our primary cohort, we identified 1689 patients with LBBB. Of these, 108 (11%) cases of dyssynchrony‐mediated cardiomyopathy were identified during a median follow‐up of 2.3 years. Among patients with RVP, chronic LBBB was associated with a 50% higher risk of developing dyssynchrony‐mediated cardiomyopathy (HR: 1.46, 95% CI: 1.15–1.85) (Table S3 and Figure S2). A similar trend was observed across pacing indications, though statistical significance was reached only in those with a pacing indication of complete heart block or atrioventricular nodal ablation (Table S3).

In an analysis of dyssynchrony‐mediated cardiomyopathy incidence by pacing modality, patients with chronic LBBB exhibited a higher risk with both leadless and transvenous pacemakers. After adjusting for baseline characteristics, statistical significance, however, was observed only in patients with transvenous devices. We did not observe a significant association in the leadless pacemaker group, likely due to limited sample size (Table S4).

### 3.4 Incident Heart Failure by LBBB QRS Duration

When stratified by LBBB QRSd, higher rates of incident systolic heart failure and dyssynchrony‐mediated cardiomyopathy were observed with increasing QRSd (Figure S3). Patients with a QRSd of ≥150 ms experienced a 14% rate of systolic heart failure and a 13% rate dyssynchrony‐mediated cardiomyopathy within 2 years following RVP implantation.

Among those with chronic LBBB, a baseline QRSd of ≥150 ms was associated with 60% increased risk of systolic heart failure (HR: 1.60, 95% CI: 1.17–2.18) and nearly a twofold increased risk of dyssynchrony‐mediated cardiomyopathy (HR: 1.81, 95% CI: 1.13–2.91) compared to patients without chronic LBBB (Table S5).

### 3.5 Incident Systolic Heart Failure, Dyssynchrony‐Mediated Cardiomyopathy, and All Cause Mortality Compared to Isolated LBBB

To evaluate the extent to which our primary and secondary outcomes were influenced by chronic LBBB itself versus the effects of RVP on chronic LBBB (i.e., dyssynchrony on dyssynchrony), we conducted a separate analysis. This analysis identified participants with chronic LBBB who did not have any cardiac device during the same time period, using a similar methodology. Incident systolic heart failure, dyssynchrony‐mediated cardiomyopathy, and mortality from the initial identification of LBBB were determined as previously described.

A total of 159,157 patients with chronic LBBB and no pacemaker were identified, with baseline characteristics detailed in Table S6. During a median follow‐up of 2.1 years, there were 8336 (5.2%) cases of new‐onset systolic heart failure, 5836 (4.6%) cases of dyssynchrony‐mediated cardiomyopathy, and 26,746 (16.8%) cases of all‐cause mortality.

**FIGURE 3:**
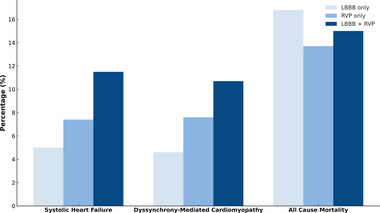
Bar chart displaying the incident rates of systolic heart failure, dyssynchrony‐mediated cardiomyopathy, and all‐cause mortality stratified by isolated LBBB (without pacemaker), RVP without LBBB, and LBBB with RVP. [Colour figure can be viewed at wileyonlinelibrary.com]

Patients with chronic LBBB without a pacemaker had lower rates of new‐onset systolic heart failure and dyssynchrony‐mediated cardiomyopathy compared to those with RVP only and those with RVP applied to pre‐existing chronic LBBB (Figure 3). The highest rates of new‐onset systolic heart failure and dyssynchrony‐mediated cardiomyopathy were observed in patients with RVP on pre‐existing or chronic LBBB, supporting the hypothesis of the “dyssynchrony on dyssynchrony” phenomenon. Mortality was notably higher in patients with chronic LBBB regardless of RVP.

## 4 Discussion

In this study, we evaluated the incidence and clinical characteristics of cardiomyopathy and all‐cause mortality in patients with normal LVEF undergoing RVP, stratified by the presence or absence of chronic LBBB. The notable findings are as follows: (1) Chronic or pre‐existing LBBB was associated with over 10% higher risk of composite mortality or systolic heart failure, predominantly driven by an increased risk of systolic heart failure. (2) Chronic LBBB was also associated with 50% higher likelihood of developing dyssynchrony‐mediated cardiomyopathy across all pacing indications, with the greatest risk observed in patients with a pacing indication of complete heart block, those concurrently undergoing atrioventricular nodal ablation and with longer baseline LBBB QRSd. (3) Patients with chronic LBBB who developed systolic heart failure were twice as likely to exhibit moderately depressed left ventricular systolic function and two times more likely to require CRT upgrades during follow‐up compared to patients without chronic LBBB.

There is limited evidence on the risk of heart failure in patients with chronic LBBB undergoing RVP. Consistent with our findings, a prior study involving 20 patients with chronic LBBB reported that RVP was associated with a fourfold increase in adjusted risks for pacemaker‐induced cardiomyopathy, even in individuals with preserved LVEF and over a relatively short follow‐up period [5]. In our study, we expanded the scope by including all‐cause systolic heart failure and mortality as primary outcomes. This approach recognizes that chronic LBBB is strongly associated with an underlying cardiomyopathic process, often linked to chronic ischemic heart disease and valvular heart disease, placing these patients at an elevated risk for heart failure and mortality beyond those related to abnormal conduction [16, 18, 19, 20, 21].

Recent advancements in pacing modalities, including leadless pacing, and conduction system pacing, have enabled a more individualized approach to device selection [22, 23]. The choice of pacing strategy is often guided by the anticipated risk of cardiomyopathy, which is influenced by baseline LVEF and expected pacing burden. Current guidelines recommend CRT via biventricular pacing or conduction system pacing for patients with a high anticipated pacing burden and reduced LVEF, though it may be considered in other cases [24]. Our findings suggest that patients with chronic LBBB may specifically benefit from CRT or conduction system pacing when a ventricular pacing indication exists, regardless of baseline LVEF. Notably, these individuals also exhibited slightly lower baseline LVEF, suggesting the presence of subclinical myocardial dysfunction that may have been exacerbated by non‐physiological pacing [25].

Emerging evidence demonstrates that conduction system pacing can normalize LBBB, particularly those with LBBB due to proximal conduction disease [23, 26, 27]. For patients with chronic LBBB requiring pacing, physiological pacing may specifically offer significant benefits by preventing both pacing‐ and LBBB‐induced cardiomyopathy, as well as cardiomyopathy potentially linked to the underlying disease process causing LBBB. These findings highlight the need for improved risk stratification beyond LVEF assessment to optimize pacing strategies and mitigate the risk of adverse remodeling in patients requiring ventricular pacing.

The natural history and pathogenesis of abnormal conduction‐induced cardiomyopathy have been described but remain poorly understood [5, 6, 12, 13, [16, 28, 29, 30]. Our analysis underscores the significant impact of RVP in individuals with baseline left ventricular dyssynchrony. Specifically, RVP in patients with chronic LBBB was associated with a risk of systolic heart failure that was twice as high as that observed in patients with LBBB without pacemakers and substantially higher compared to those with RVP without LBBB. These findings highlight the compounded risk of dyssynchronous pacing applied to an already dyssynchronous ventricle, emphasizing that the risk is not solely attributable to LBBB or RVP individually [6, 7, 8].

This study has several limitations. First, it relied on ICD‐10‐CM and PCS codes to identify primary exposures and outcomes, which may be subject to diagnostic inaccuracies. Nevertheless, these codes are well‐validated, have been utilized in prior research, and enhance the credibility of our findings and analyses. Second, while data on LVEF, NT‐proBNP levels, and medication use following implantation were available, the timing of these measurements was not clearly defined. This ambiguity could introduce bias, particularly if values were collected after CRT upgrades or changes in device management, such as adjustments to pacing modes favoring intrinsic conduction. Third, despite employing propensity score matching to account for baseline differences, residual confounding may remain due to unmeasured factors, such as specific clinical or procedural variables, that could affect heart failure risk.

Additionally, the absence of granular data on device‐specific characteristics, including pacing modality, pacing burden, and lead location, limits the ability to fully explore their role in heart failure development. Moreover, the diagnosis of LBBB based on ECG criteria alone does not confirm the presence of mechanical dyssynchrony, as intraventricular conduction delay may mimic LBBB [31]. True mechanical dyssynchrony can only be confirmed with echocardiographic assessment. Given that the absence of exclusion criteria does not equate to confirmed dyssynchrony, we acknowledge that our findings reflect presumed dyssynchrony‐mediated cardiomyopathy rather than definitively established dyssynchrony‐induced disease. Future studies should incorporate more detailed echocardiographic and device‐related data to refine patient selection and optimize pacing strategies, particularly in populations with conduction abnormalities such as chronic LBBB.

## 5 Conclusion

Among patients undergoing RVP implantation with normal LVEF, chronic LBBB is associated with a higher risk of systolic heart failure and moderately depressed left ventricular systolic function, highlighting the need for improved risk stratification beyond LVEF assessment. Future studies should explore whether patients with chronic LBBB may benefit from upfront CRT or conduction system pacing to mitigate this risk.

## Author Contributions

Temidayo A. Abe contributed to review and editing, writing the original draft, supervision, and methodology. Favour Markson contributed to review and editing, statistical analysis, and methodology. Daniel J. Friedman contributed to review and editing, supervision, and methodology. Larry R. Jackson II contributed to review and editing, supervision, and methodology.

## Disclosure

The remaining authors have no relevant disclosures.

## Conflicts of Interest

The authors declare no conflicts of interest.

## Supporting information




**Supplemental Table 1**: International Classification of Diseases and Clinical Classification Software Codes Used in Identification of Clinical Variables.
**Supplemental Table 2**: Clinical Outcomes After Pacemaker Implantation in Patients with and without Preexisting LBBB Before Propensity Matching.
**Supplemental Table 3**: Incident Dyssynchrony‐Mediated Cardiomyopathy Post‐Pacemaker Implantation in Patients with and without Preexisting LBBB, Stratified by Pacing Indication.
**Supplemental Table 4**: Incident Dyssynchrony‐Mediated Cardiomyopathy Post‐Pacemaker Implantation in Patients with and without Preexisting LBBB, Stratified by Pacing Modality.
**Supplemental Table 5**: Incident Systolic Heart Failure and Dyssynchrony‐Mediated Cardiomyopathy Post‐Pacemaker Implantation in Patients with and without Preexisting LBBB, Stratified by LBBB QRS Duration.
**Supplemental Table 6**: Baseline Characteristics of Patients Isolated Left Bundle Branch Block without Pacemaker.
**Supplemental Figure 1**: Propensity density score before and after matching of patients undergoing RVP with versus without LBBB.
**Supplemental Figure 2**: Kaplan‐Meier survival curve depicting freedom from dyssynchrony‐mediated cardiomyopathy (DMC) over time (in days post‐implantation) in patients with RVP with chronic LBBB compared to those without chronic LBBB. The curves for the chronic LBBB group (purple), and no LBBB (green).
**Supplemental Figure 3**: Incident systolic heart failure and dyssynchrony‐mediated cardiomyopathy among patients with RVP with chronic LBBB stratified by LBBB QRS duration.

## Data Availability

This study leveraged data from the TriNetX Analytics Network database. The database is publicly available to licensed users through the TriNetX platform

## References

[1] J. F. Huizar , K. Kaszala , A. Tan , et al., “Abnormal Conduction‐Induced Cardiomyopathy,” Journal of the American College of Cardiology 81 (2023): 1192–1200, 10.1016/j.jacc.2023.01.040. 36948737 PMC10715964

[2] G. Kaye , J. Y. Ng , S. Ahmed , D. Valencia , D. Harrop , and A. C. T. Ng , “The Prevalence of Pacing‐Induced Cardiomyopathy (PICM) in Patients With Long Term Right Ventricular Pacing − Is It a Matter of Definition?,” Heart, Lung and Circulation 28 (2019): 1027–1033, 10.1016/j.hlc.2018.05.196. 30017634

[3] E. Ebrille , C. V. DeSimone , V. R. Vaidya , A. A. Chahal , V. T. Nkomo , and S. J. Asirvatham , “Ventricular Pacing—Electromechanical Consequences and Valvular Function,” Indian Pacing and Electrophysiology Journal 16 (2016): 19–30, 10.1016/j.ipej.2016.02.013. 27485561 PMC4936653

[4] S. Saba , H. Mehdi , M. A. Mathier , M. Z. Islam , G. Salama , and B. London , “Effect of Right Ventricular Versus Biventricular Pacing on Electrical Remodeling in the Normal Heart,” Circulation: Arrhythmia and Electrophysiology 3 (2010): 79–87, 10.1161/CIRCEP.109.889741. 20042767 PMC2834320

[5] S. W. Cho , H. B. Gwag , J. K. Hwang , et al., “Clinical Features, Predictors, and Long‐Term Prognosis of Pacing‐Induced Cardiomyopathy,” European Journal of Heart Failure 21, no. 5 (2019): 643–651, 10.1002/ejhf.1427. 30734436

[6] S. Khurshid , A. E. Epstein , R. J. Verdino , et al., “Incidence and Predictors of Right Ventricular Pacing‐Induced Cardiomyopathy,” Heart Rhythm 11 (2014): 1619–1625, 10.1016/j.hrthm.2014.05.040. 24893122

[7] S. A. Lee , M. J. Cha , Y. Cho , I. Y. Oh , E. K. Choi , and S. Oh , “Paced QRS Duration and Myocardial Scar Amount: Predictors of Long‐Term Outcome of Right Ventricular Apical Pacing,” Heart and Vessels 31 (2016): 1131–1139, 10.1007/s00380-015-0707-8. 26142378

[8] J. Y. Chin , K. W. Kang , S. H. Park , et al., “Pre‐Implant Global Longitudinal Strain as an Early Sign of Pacing‐Induced Cardiomyopathy in Patients With Complete Atrioventricular Block,” Echocardiography 38 (2021): 175–182, 10.1111/echo.14942. 33406280 PMC7986095

[9] T. A. Boyle , N. V. K. Pothineni , M. Austin , et al., “Incidence and Predictors of Pacing‐Induced Right Ventricular Cardiomyopathy,” Circulation: Arrhythmia and Electrophysiology 17 (2024): e013070, 10.1161/CIRCEP.124.013070. 39324275

[10] O. A. Smiseth and J. M. Aalen , “Mechanism of Harm From Left Bundle Branch Block,” Trends in Cardiovascular Medicine 29 (2019): 335–342, 10.1016/j.tcm.2018.10.012. 30401603

[11] K. Russell , M. Eriksen , L. Aaberge , et al., “A Novel Clinical Method for Quantification of Regional Left Ventricular Pressure–Strain Loop Area: A Non‐Invasive Index of Myocardial Work,” European Heart Journal 33 (2012): 724–733, 10.1093/eurheartj/ehs016. 22315346 PMC3303715

[12] X. Wang , B. Ge , C. Miao , et al., “Beyond Conduction Impairment: Unveiling the Profound Myocardial Injury in Left Bundle Branch Block,” Heart Rhythm 21 (2024): 1370–1379, 10.1016/j.hrthm.2024.03.012. 38490601

[13] C. L. Grines , T. M. Bashore , H. Boudoulas , S. Olson , P. Shafer , and C. F. Wooley , “Functional Abnormalities in Isolated Left Bundle Branch Block. The Effect of Interventricular Asynchrony,” Circulation 79 (1989): 845–853, 10.1161/01.CIR.79.4.845. 2924415

[14] J.‐J. Blanc , M. Fatemi , V. Bertault , F. Baraket , and Y. Etienne , “Evaluation of Left Bundle Branch Block as a Reversible Cause of Non‐Ischaemic Dilated Cardiomyopathy With Severe Heart Failure. A New Concept of Left Ventricular Dyssynchrony‐Induced Cardiomyopathy,” Europace 7 (2005): 604–610, 10.1016/j.eupc.2005.06.005. 16216764

[15] C. Vaillant , R. P. Martins , E. Donal , et al., “Resolution of Left Bundle Branch Block–Induced Cardiomyopathy by Cardiac Resynchronization Therapy,” Journal of the American College of Cardiology 61 (2013): 1089–1095, 10.1016/j.jacc.2012.10.053. 23352778

[16] W. Barake , C. M. Witt , V. R. Vaidya , and Y.‐M. Cha , “Incidence and Natural History of Left Bundle Branch Block Induced Cardiomyopathy,” Circulation: Arrhythmia and Electrophysiology 12, no. 9 (2019): e007393, 10.1161/CIRCEP.119.007393. 31510788

[17] M. B. Palchuk , J. W. London , D. Perez‐Rey , et al., “A Global Federated Real‐World Data and Analytics Platform for Research,” JAMIA Open 6 (2023): ooad035, 10.1093/jamiaopen/ooad035. 37193038 PMC10182857

[18] N. Y. Tan , C. M. Witt , J. K. Oh , and Y.‐M. Cha , “Left Bundle Branch Block: Current and Future Perspectives,” Circ Arrhythm Electrophysiol 13, no. 4 (2020), e008239, 10.1161/CIRCEP.119.008239. 32186936

[19] N. E. Lebtahi , J. C. Stauffer , and A. B. Delaloye , “Left Bundle Branch Block and Coronary Artery Disease: Accuracy of Dipyridamole Thallium‐201 Single‐Photon Emission Computed Tomography in Patients With Exercise Anteroseptal Perfusion Defects,” Journal of Nuclear Cardiology 4 (1997): 266–273, 10.1016/s1071-3581(97)90103-3. 9278872

[20] I. J. Neeland , M. C. Kontos , and J. A. de Lemos , “Evolving Considerations in the Management of Patients With Left Bundle Branch Block and Suspected Myocardial Infarction,” Journal of the American College of Cardiology 60 (2012): 96–105, 10.1016/j.jacc.2012.02.054. 22766335 PMC3402162

[21] M. Sugiura , R. Okada , S. Okawa , and H. Shimada , “Pathohistological Studies on the Conduction System in 8 Cases of Complete Left Bundle Branch Block,” Japanese Heart Journal 11 (1970): 5–16, 10.1536/ihj.11.5. 5309105

[22] A. I. Vouliotis , P. R. Roberts , P. Dilaveris , K. Gatzoulis , A. Yue , and K. Tsioufis , “Leadless Pacemakers: Current Achievements and Future Perspectives,” European Cardiology 18 (2023): e49, doi:10.15420/ecr.2022.32. 37655133 PMC10466270

[23] P. Vijayaraman , M. G. Chelu , K. Curila , et al., “Cardiac Conduction System Pacing,” JACC: Clinical Electrophysiology 9 (2023): 2358–2387, 10.1016/j.jacep.2023.06.005. 37589646

[24] M. K. Chung , K. K. Patton , C.‐P. Lau , et al., “2023 HRS/APHRS/LAHRS Guideline on Cardiac Physiologic Pacing for the Avoidance and Mitigation of Heart Failure,” Heart Rhythm 20 (2023): e17–e91, 10.1016/j.hrthm.2023.03.1538. 37283271 PMC11062890

[25] A. Zegard , O. Okafor , J. de Bono , et al., “Prognosis of Incidental Left Bundle Branch Block,” EP Europace 22 (2020): 956–963, 10.1093/europace/euaa008. 32285097

[26] G. A. Upadhyay , T. Cherian , D. Y. Shatz , et al., “Intracardiac Delineation of Septal Conduction in Left Bundle‐Branch Block Patterns,” Circulation 139 (2019): 1876–1888, 10.1161/CIRCULATIONAHA.118.038648. 30704273

[27] F. Salden , J. Luermans , S. W. Westra , et al., “Short‐Term Hemodynamic and Electrophysiological Effects of Cardiac Resynchronization by Left Ventricular Septal Pacing,” Journal of the American College of Cardiology 75 (2020): 347–359, 10.1016/j.jacc.2019.11.040. 32000945

[28] S. S. Ponnusamy and P. Vijayaraman , “Left Bundle Branch Block–Induced Cardiomyopathy,” JACC: Clinical Electrophysiology 7 (2021): 1155–1165, 10.1016/j.jacep.2021.02.004. 33812829

[29] G. Moe , “Pacing‐Induced Heart Failure: A Model to Study the Mechanism of Disease Progression and Novel Therapy in Heart Failure,” Cardiovascular Research 42 (1999): 591–599, 10.1016/S0008-6363(99)00032-2. 10533598

[30] F. M. Merchant , M. H. Hoskins , D. L. Musat , et al., “Incidence and Time Course for Developing Heart Failure With High‐Burden Right Ventricular Pacing,” Circulation: Cardiovascular Quality and Outcomes 10, no. 6 (2017): e003564, 10.1161/CIRCOUTCOMES.117.003564. 28630373

[31] J. S. Treger , A. B. Allaw , P. Razminia , et al., “A Revised Definition of Left Bundle Branch Block Using Time to Notch in Lead I,” JAMA Cardiology 9 (2024): 449, 10.1001/jamacardio.2024.0265. 38536171 PMC10974693

