# Supplementary material for: Characteristics of Cardiomyopathy in Patients With Chronic Left Bundle Branch Block Undergoing Right Ventricular Pacing
Source: Pacing Clin Electrophysiol. 2025 Aug 8;48(9):973–80. doi: 10.1111/pace.70020 (PMC12439233; doi:10.1111/pace.70020)
Supplement: Supplementary file 1 — Supplemental Table 1: International Classification of Diseases and Clinical Classification Software Codes Used in Identification of Clinical Variables. Supplemental Table 2: Clinical Outcomes After Pacemaker Implantation in Patients with and without Preexisting LBBB Before Propensity Matching. Supplemental Table 3: Incident Dyssynchrony‐Mediated Cardiomyopathy Post‐Pacemaker Implantation in Patients with and without Preexisting LBBB, Stratified by Pacing Indication. Supplemental Table 4: Incident Dyssynchrony‐Mediated Cardiomyopathy Post‐Pacemaker Implantation in Patients with and without Preexisting LBBB, Stratified by Pacing Modality. Supplemental Table 5: Incident Systolic Heart Failure and Dyssynchrony‐Mediated Cardiomyopathy Post‐Pacemaker Implantation in Patients with and without Preexisting LBBB, Stratified by LBBB QRS Duration. Supplemental Table 6: Baseline Characteristics of Patients Isolated Left Bundle Branch Block without Pacemaker. Supplemental Figure 1: Propensity density score before and after matching of patients undergoing RVP with versus without LBBB. Supplemental Figure 2: Kaplan‐Meier survival curve depicting freedom from dyssynchrony‐mediated cardiomyopathy (DMC) over time (in days post‐implantation) in patients with RVP with chronic LBBB compared to those without chronic LBBB. The curves for the chronic LBBB group (purple), and no LBBB (green). Supplemental Figure 3: Incident systolic heart failure and dyssynchrony‐mediated cardiomyopathy among patients with RVP with chronic LBBB stratified by LBBB QRS duration. [file PACE-48-973-s001.docx]

Supplemental Table 1: International Classification of Diseases and Clinical Classification Software Codes Used in Identification of Clinical Variables.

| Procedure | ICD-10 PCS codes | CPT codes |
| --- | --- | --- |
| AV-nodal ablation | - | 93650 |
| CRT | 02H43KZ, 02H43JZ, 02H40JZ, 02H40KZ,  02HL0JZ, 02HL0KZ, 02HL3JZ, 02HL3KZ, 02HL4JZ, 02HL4KZ | 33224,33225,33226 |
| Leadless pacemaker insertion | 02HK3NZ | 33274 |
| Transvenous right ventricular lead/Dual Chamber leads | 0JH606Z, 02HK3JZ, 02HK4JZ, 02H63JZ, | 33206, 33207, 33208, 33214. |
|  | | |
| Diagnosis | ICD-10 CM codes |  |
| Atrial fibrillation/flutter | I48.xx |  |
| Atrioventricular block | I44.1 |  |
| Complete heart block | I44.2 |  |
| Diabetes mellitus | E08 – E13 |  |
| Hypertension | I10 – I1A |  |
| Hypothyroidism | E03.xx |  |
| Hyperthyroidism | E05.xx |  |
| LBBB | I44.7 |  |
| Prior coronary interventions | Z95.5, Z95.1 |  |
| Prior myocardial infarction | I12.2 |  |
| Sinus node dysfunction | I49.5 |  |
|  | | |
| Laboratory | Definition (LOINC codes) |  |
| BMI | 39156-5 |  |
| NT-BNP | 33762-6 |  |
| Creatinine | 14682-9 |  |
| Hemoglobin A1c | 4584-4 |  |
| Left Ventricular Ejection Fraction | 10230-1 |  |
|  | | |
| Medications | Definition (VA class codes)/RxNorm |  |
| Beta-blockers | CV100 |  |
| ACE-inhibitors | CV800, CV805 |  |
| MRA | CV704 |  |
| ARNi | 1656328* |  |
| SGLT2-i | 1545653*, 1488564* |  |

 ARNi-Angiotensin Receptor/Neprilysin inhibitor, AV-Atrioventricular, BMP- Body mass index, NT-BNP- N-Terminal pro Brain Natriuretic Peptide, CPT-Current Procedural Terminology, CRT- Chronic Resynchronization Therapy, LBBB- Left bundle branch block, LOINC codes – Logical Observation Identifier Names and Codes, MRA- mineralocorticoid receptor antagonist, SGLT2i- Sodium-Glucose Cotransporter 2 Inhibitors, VA class codes -Veteran Affairs Drug classification

Supplemental Table 2: Clinical Outcomes After Pacemaker Implantation in Patients with and without Preexisting LBBB Before Propensity Matching

|  | RVP | |  |  |
| --- | --- | --- | --- | --- |
|  | LBBB  (n=3,916) | No LBBB  (n=66,610) | HR (95% CI) | P- Value |
| Composite mortality or heart failure | 24.0 | 19.0 | 1.35 (1.25-1.45) | <0.001 |
| New onset systolic heart failure | 11.5 | 7.4 | 1.64 (1.48-1.82) | <0.001 |
| All-cause mortality | 15.0 | 13.7 | 1.16 (1.02-1.22) | 0.018 |

Data are presented in percentages. The hazard ratio, 95% confidence interval (CI), and corresponding P-values for comparisons between the two groups are included. Abbreviations: LBBB- left bundle branch block; CI-confidence interval; HR; hazard ratio; RVP-right ventricular pacemaker.

Supplemental Table 3: Incident Dyssynchrony-Mediated Cardiomyopathy Post-Pacemaker Implantation in Patients with and without Preexisting LBBB, Stratified by Pacing Indication

|  | RVP | |  |  |
| --- | --- | --- | --- | --- |
|  | LBBB | No LBBB | HR (95% CI) | P- Value |
| Overall  N=1,688 | 10.7 | 7.6 | 1.46 (1.15-1.82) | 0.002 |
| Sinus node dysfunction  N=477 | 13.4 | 10.3 | 1.35 (0.91-2.01) | 0.133 |
| Atrioventricular block  N= 494 | 11.1 | 7.9 | 1.49 (0.95-2.25) | 0.079 |
| Complete heart block or AVN ablation  N=976 | 12.1 | 9.3 | 1.34 (1.00-1.79) | 0.048 |

N represents the total number in the study cohort after 1:1 propensity score matching overall and by pacing indication. Data are presented in percentages. The hazard ratio, 95% confidence interval (CI), and corresponding P-values for comparisons between the two groups are included. Abbreviations: AVN-atrioventricular nodal; CI-confidence interval; HR; hazard ratio; LBBB- left bundle branch block; RVP-right ventricular pacemaker.

Supplemental Table 4: Incident Dyssynchrony-Mediated Cardiomyopathy Post-Pacemaker Implantation in Patients with and without Preexisting LBBB, Stratified by Pacing Modality

| Transvenous Pacemaker | | | | Leadless Pacemaker | | | |
| --- | --- | --- | --- | --- | --- | --- | --- |
| LBBB  (1,548) | No LBBB  (22,720) | HR  (95% CI) | P- Value | LBBB  (107) | No LBBB  (1,598) | HR  (95% CI) | P- Value |
| 10.5 | 6.4 | 1.32 (1.04-1.69) | <0.001 | 9.3 | 5.6 | 0.95  (0.38-2.51) | 0.99 |

Data are presented in percentages crude rates (unadjusted) and adjusted hazard ratio after propensity score matching. The hazard ratio, 95% confidence interval (CI), and corresponding P-values for comparisons between the two groups are included. Abbreviations: CI-confidence interval; HR; hazard ratio; LBBB- left bundle branch block.

Supplemental Table 5: Incident Systolic Heart Failure and Dyssynchrony-Mediated Cardiomyopathy Post-Pacemaker Implantation in Patients with and without Preexisting LBBB, Stratified by LBBB QRS Duration

|  | HR  (95% CI) | P-Value |
| --- | --- | --- |
| Systolic heart failure |  |  |
| LBBB QRSd  >150 ms | 1.60 (1.17-2.18) | 0.003 |
| Dyssynchrony-Mediated Cardiomyopathy |  |  |
| LBBB QRSd  >150 ms | 1.81 (1.13-2.91) | 0.014 |

The adjusted hazard ratio after propensity score matching in patients with LBBB and QRSd > 150 ms compared to no LBBB (REF): CI-confidence interval; HR; hazard ratio; LBBB- left bundle branch block.

Supplemental Table 6: Baseline Characteristics of Patients Isolated Left Bundle Branch Block without Pacemaker

| Characteristics | N=20,388 |
| --- | --- |
| Age, mean ± SD | 70±14 |
| Sex |  |
| Male | 42 |
| Female | 54 |
| Race |  |
| White | 72 |
| Black | 5.8 |
| Asian | 1.9 |
| Others | 16.1 |
| Ethnicity |  |
| Hispanic or Latino | 42.7 |
| Comorbidities |  |
| Diabetes mellitus | 16.6 |
| Hypertension | 45.2 |
| Hypothyroidism | 10.6 |
| Hyperthyroidism | 1.2 |
| Ischemic heart disease | 17.3 |
| Prior PCI | 2.3 |
| Prior CABG | 2.2 |
| Atrial fibrillation/flutter | 8.7 |
| Body mass index, mean ± SD | 28.9± 6 |
| Laboratory |  |
| Creatinine, mean ± SD | 1.13± 4 |
| Hemoglobin A1c, mean ± SD | 6.33± 2 |
| LVEF, mean ± SD | 61.7± 7 |
| NT-proBNP pg/ml, mean ± SD | 252± 874 |

Data are presented as mean ± standard deviation (SD) for continuous variables and percentages for categorical variables. Abbreviations: CABG – coronary artery bypass graft; CVD – cardiovascular disease; LBBB – left bundle branch block; LVEF – left ventricular ejection fraction; NT-proBNP – N-terminal pro-B type natriuretic peptide; PCI – percutaneous coronary intervention; PS-propensity score; RVP-right ventricular pacemaker; SD-standard deviation.


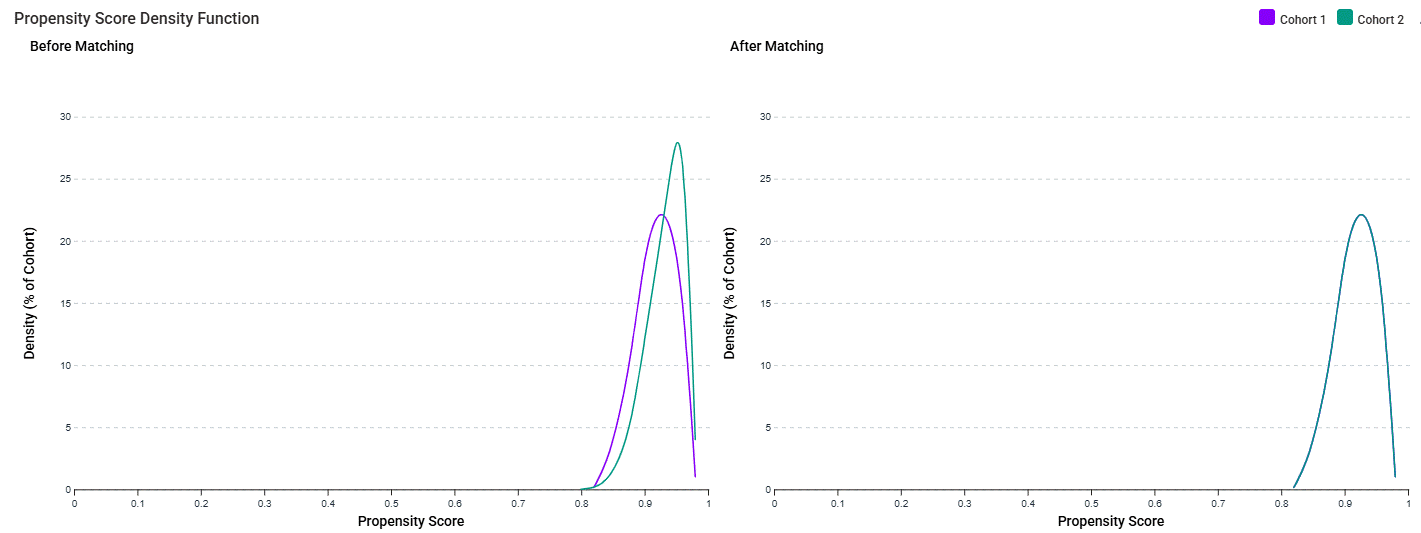


Supplemental Figure 1: Propensity density score before and after matching of patients undergoing RVP with versus without LBBB.

Supplemental Figure 2: Kaplan-Meier survival curve depicting freedom from dyssynchrony-mediated cardiomyopathy (DMC) over time (in days post-implantation) in patients with RVP with chronic LBBB compared to those without chronic LBBB. The curves for the chronic LBBB group (purple), and no LBBB (green).

Supplemental Figure 3: Incident systolic heart failure and dyssynchrony-mediated cardiomyopathy among patients with RVP with chronic LBBB stratified by LBBB QRS duration.
